# The Aggradational Successions of the Aniene River Valley in Rome: Age Constraints to Early Neanderthal Presence in Europe

**DOI:** 10.1371/journal.pone.0170434

**Published:** 2017-01-26

**Authors:** Fabrizio Marra, Piero Ceruleo, Luca Pandolfi, Carmelo Petronio, Mario F. Rolfo, Leonardo Salari

**Affiliations:** 1 Istituto Nazionale di Geofisica e Vulcanologia,Via di Vigna Murata, Roma, Italy; 2 Freelance, Tivoli (Roma), Italy; 3 Dipartimento di Scienze, sezione di Geologia, Università degli Studi "Roma Tre", Largo San Leonardo Murialdo, Roma, Italy; 4 Dipartimento di Scienze della Terra, Sapienza, Università di Roma, Piazzale Aldo Moro, Roma, Italy; 5 Dipartimento di Storia, Cultura e Società, Università di Roma "Tor Vergata", Via Columbia, Roma, Italy; Max Planck Institute for the Science of Human History, GERMANY

## Abstract

We revise the chronostratigraphy of several sedimentary successions cropping out along a 5 km-long tract of the Aniene River Valley in Rome (Italy), which yielded six hominin remains previously attributed to proto- or archaic Neanderthal individuals, as well as a large number of lithic artefacts showing intermediate characteristics somewhere between the local Acheulean and Mousterian cultures. Through a method of correlation of aggradational successions with post-glacial sea-level rises, relying on a large set of published ^40^Ar/^39^Ar ages of interbedded volcanic deposits, we demonstrate that deposition of the sediments hosting the human remains spans the interval 295–220 ka. This is consistent with other well constrained ages for lithic industries recovered in England, displaying transitional features from Lower to Middle Paleolithic, suggesting the appearance of Mode 3 during the MIS 9-MIS 8 transition. Moreover, the six human bone fragments recovered in the Aniene Valley should be regarded as the most precisely dated and oldest hominin remains ascribable to Neanderthal-type individuals in Europe, discovered to date. The chronostratigraphic study presented here constitutes the groundwork for addressing re-analysis of these remains and of their associated lithic industries, in the light of their well-constrained chronological picture.

## Introduction

**Fig 1:**
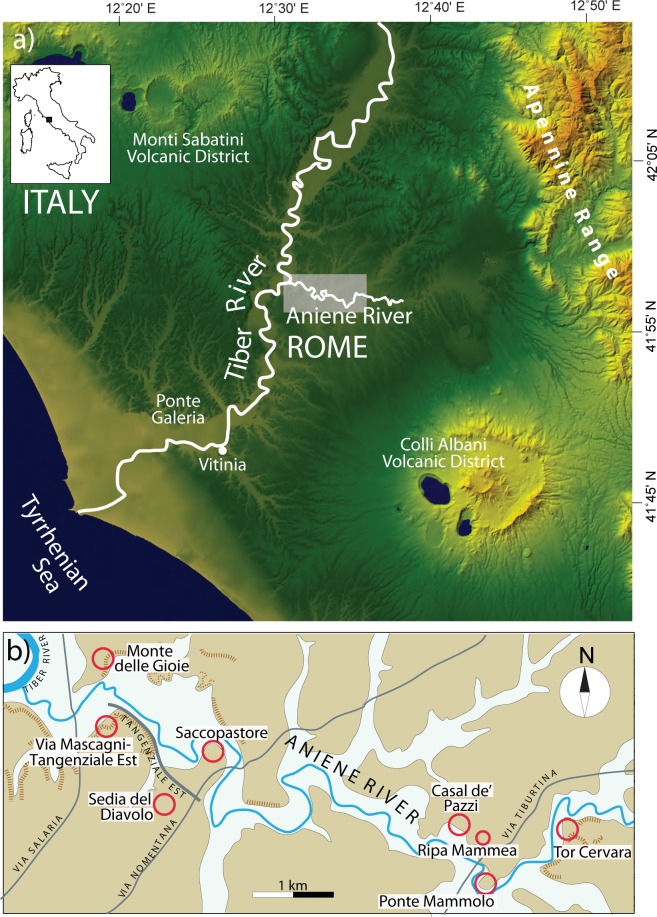
Location map showing the investigated geologic sections of the Aniene River Valley. a) Digital Elevation Map (DEM) TINITALY/01 square WA 6570, used with permission of the Istituto Nazionale di Geofisica e Vulcanologia, Rome (S1 File); b) Location map showing the investigated sites.

Six hominin remains have been recovered since the middle of the 19th century from the Middle Pleistocene sedimentary successions cropping out at four locations (Saccopastore, Casal de’ Pazzi, Ponte Mammolo, Sedia del Diavolo) along a short tract (~5 km) of the Aniene River Valley in northern Rome (Fig 1), the most relevant ones being two skulls of *Homo neanderthalensis* found in the years 1929 [1] and 1935 [2]. An age ranging from130,000–80,000 years BP was attributed to these skulls, occurring within fluvial gravel beds exposed by quarry excavation at the Saccopastore site, until a recent study [3] demonstrated that the sedimentary succession in which they were recovered dates back to 250,000 years BP, providing the oldest Italian evidence of *H*. *neanderthalensis*. Previous estimation of age was based on the sedimentary deposits being classified as a fluvial terrace of the last interglacial stage, an estimate which was made at the time of the discovery [4, 5, 6]. In contrast, [3] have shown that the fluvial-lacustrine deposits of Saccopastore correlate with the aggradational succession deposited within the Tiber and Aniene river valleys [7] in response to the post-glacial sea-level rise during glacial termination 3 (T-3), at the onset of Marine Isotopic Stage (MIS) 7.

Besides correlating the sedimentary deposits by means of sedimentological, stratigraphical and geomorphological evidence, the authors have shown the compatibility of the vertebrate fossil assemblages found in the same sedimentary deposits hosting the two skulls, with other Italian faunal assemblages attributed to the MIS 7 Vitinia Faunal Unit (= FU; [8, 9]), and recovered in other sedimentary successions in the area of Rome (e.g. Torre in Pietra 'level d' [10], Casal de' Pazzi ([11]), Vitinia type-locality [12]. Compatibility has also been shown with the faunal assemblages recovered within the Monte delle Gioie and Sedia del Diavolo sedimentary deposits [4, 5], which are older than 285.000 years BP and correlate with aggradational succession of sub-stage 8.5 [13]. In the present work we demonstrate this correlation further, through the re-examination of several specimens of vertebrate remains originally recovered in Saccopastore ([1, 5] and references therein) and held at the "Museo Nazionale Preistorico Etnografico Luigi Pigorini" in Rome. Among these, we have determined the presence of *Dama dama tiberina*, a marker of the Vitinia FU, whose occurrence in the faunal assemblages of Italy is limited to MIS 8.5 and MIS 7 ([13], and references therein), implying an age comprised between 295 and 200 ka for the sedimentary succession of Saccopastore. Moreover, by refining correlation with the sea-level rises which occurred in this time span, we assign ages of 245 ka and 220 ka to the Neanderthal skulls recovered within the lowest and the uppermost gravel layer at Saccopastore, respectively.

The archaic Neanderthal features of these skulls have been repeatedly noted (e.g.: [14]). The other hominin remains recovered at the sites of the Aniene Valley shown in Fig 1 were variably described as showing the features of archaic *Homo* (a parietal found at Casal de' Pazzi; [15]), as proto-Neanderthal (a femur diaphysis from Ponte Mammolo [16]), or showing affinity with typical Neanderthal individuals (a femur diaphysis and a second metatarsus from Sedia del Diavolo [17]). Similarly, the lithic industries associated with these remains, or occurring in the sedimentary successions at nearby locations (i.e.: Monte delle Gioie [18], Ripa Mammea [19]; Fig 1), were attributed to the Early Middle- (e.g.: "proto-Pontinian" [18]) through Middle-Palaeolithic (i.e.: Mousterian, locally referred to as "Pontinian" [20]), following nomenclature and criteria in use at the time of their discovery.

The focus of the present study is to assess the age of the abovementioned hominin remains and of the associated lithic artefacts, in order to compare it to that of the earliest direct Neanderthal evidence and to that of the techno-cultural complexes associated with its spread throughout Europe. These events have questionable dates depending on the different dating methods used and on their attribution to the hominin species as well as to the technological culture. In particular, dates for the oldest direct Neanderthal evidence in Europe are associated with many uncertainties and date as far back as 253 +53/-37 ka [21]. Similarly, several age constraints have been reported for the transition between the Lower and the Middle Palaeolithic, which refer it to the MIS 9–8 transition spanning 320–270 ka [22, 23, 24, 25, 26, 27, 28].

By studying the sedimentologic and stratigraphic features of the successions in which these remains were found, and by providing a comparative review of the associated faunal and lithic assemblages, we demonstrate that the deposition of these successions spans the time interval comprised between 295 and 220 ka, and that the hominin remains and the associated lithic industries recovered within these sediments should be regarded as the oldest direct and indirect evidence of Neanderthal in Italy, and possibly in Europe (the hominins), discovered to date.

## Materials and Methods

### Permits and repositories

The Saccopastore collection is housed in the "Museo Nazionale Preistorico Etnografico Luigi Pigorini" in Rome. Permission to study and take photographs of the materials was obtained from the Director of the Polo Museale del Lazio, Edith Gabrielli, and from the Director of the Pigorini Museum, Francesco Rubat Borel.

Permission to study and take photographs of the Casal de' Pazzi site was provided by the Director of the Casal de' Pazzi Museum, Patrizia Gioia.

No specific permission was required to perform the geologic surveys at Monte delle Gioie, Ponte Mammolo, and Tor Cervara locations because they are on public soil and the investigators (F.M., C.P and M.F.R.) are public employees in Rome at the Istituto Nazionale di Geofisica e Vulcanologia, Università La Sapienza and Università Tor Vergata, respectively.

Permission from copyright holders of a few figures is provided in the S1 File.

### Geologic investigations

**Fig 2:**
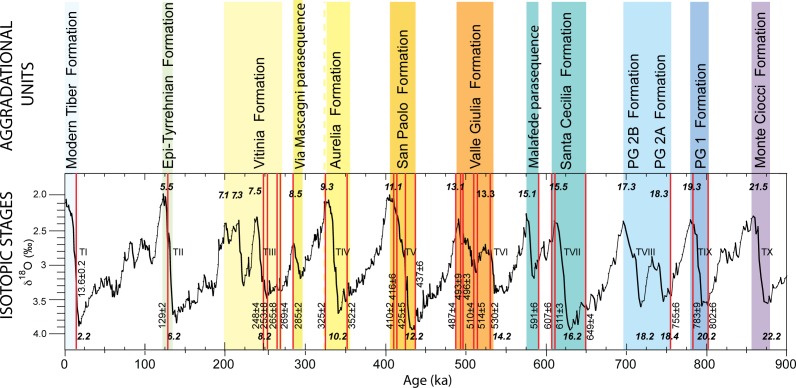
A series of geochronologically constrained aggradational phases (colored vertical boxes), corresponding to the same number of formally introduced sedimentary successions (Formations) in the area of Rome, has been reconstructed and directly correlated with post-glacial sea-level rises (glacial terminations TI—TX) and the corresponding marine isotopic stages (MISs) [3, 7, 31, 32, 33, 34]. Red vertical bars are the ^40^Ar/^39^Ar weighted mean ages (in ka) from abovementioned literature constraining the aggradational phases and providing their correlation with the Oxygen isotope curve by [44].

We correlate the sedimentary deposits cropping out at the investigated sections with the geochronologically constrained aggradational successions, which have been defined in this region (Fig 2), by integrating a review of the literature produced since the beginning of the last century with previous chronostratigraphic work at the geologic section of Via Mascagni/Tangenziale Est and with new field data achieved for the present study at the Monte delle Gioie, Casal de' Pazzi, Ponte Mammolo and Tor Cervara locations (Fig 1).

Field investigations are conducted following the basic principles of geologic survey, including visual lithostratigraphic analyses of the outcropping rocks to describe their texture, granulometry, coherence, color and mineralization, as well as presence of vegetal, faunal and anthropic material. A detailed sampling of the strata is also performed in order to make more in depth petrographic, geochemical, and geochronological analyses, when necessary. Thickness and relative elevation of the strata are accurately recorded and refer to available geodetic elevation points. A complete photographic documentation is also produced to provide accurate record of the stratigraphic setting.

### Chronostratigraphic correlations

When applicable, we use direct field observations to interpret and geochronologically constrain the stratigraphic schemes reported in literature for the sections where other hominin and lithic artefacts were recovered, in analogy with the chronostratigraphic approach used by [3] in order to revise the age of the deposits occurring in Saccopastore. This methodological approach relies on the concept of aggradational succession deposited in response to sea-level rise during the glacial terminations. This has been defined and applied to identify a suite of sedimentary successions in the near-costal and in the coastal areas of Rome, whose ages have been constrained by means of ^40^Ar/^39^Ar dating of interbedded volcanic layers and paleomagnetic investigation of clay intervals, allowing for their correlation with the Marine Isotopic Stages (MIS) [7, 29, 30, 31, 32, 33, 34]. Generally, the aggradational successions recognized in Rome display a coarse gravel layer at the base of each section, followed by a relatively thin sand horizon, which grades upward into a several-meter-thick package of sandy silt and clay deposits. In particular, a conceptual model was recently refined [35], in which gravel-clay transitions serve as a proxy for glacial terminations. The aggradational successions in the area of Rome are therefore a discontinuous stratigraphic record, composed of a succession of ten major aggradational units deposited during MIS 21 to MIS 1 by the Tiber River and its tributaries in the areas ranging from near coastal to coastal. In addition, several minor successions are associated with the more pronounced sea-level oscillations corresponding to sub-stages of the Oxygen isotope timescale, representing the physical remnants of the same number of glacio-eustatic cycles in this time span. The corresponding sedimentary deposits have been represented by formation names [7], as shown in Fig 2. Based on the definition of stratigraphic sequence (e.g.: [36, 37]) all the recognized aggradational successions represent the innermost part of high-frequency sequences in relation to glacio-eustasy. These sedimentary deposits have been exposed by continuous uplift affecting the coastal area [38, 39]. The adopted methodology is therefore similar to that used in recent years in England to correlate fluvial terraces with the MIS's in order to provide precise geochronologic constraints to the lithic assemblages recovered in the sedimentary successions of the Solent River [23, 40, 41]. In contrast, other worldwide evidence of terrace formation in response to erosional/sedimentary isostasy (e.g. [42]), [38] has suggested a main tectonic origin for the coastal uplift in the area of Rome, based on the identification of two early pulses of uplift around 800 and 600 ka. These were coincident with the beginning of the main eruptive phases at the Monti Sabatini and Colli Albani districts, suggesting a link with the back-arc geodynamic context of this region [43]. Therefore, the stratigraphic record of each glacio-eustatic cycle occurs at different elevation, depending on the absolute sea-level during each glacial/interglacial period and on the amount of uplift experienced since deposition, offering further geometric criteria for its identification.

## Geological Setting

The area of Rome is located on the Tyrrhenian Sea margin of Central Italy, SW of the Apennine mountain range, and is crossed by the Tiber and Aniene Rivers (Fig 1). The city center is just downstream of the confluence, whereas the urban area extends onto a hilly landscape, cut by the hydrographic networks of these rivers. Two volcanic districts that were active in the time span 802–36 ka [45, 46], the Colli Albani and the Monti Sabatini, are located SE and NW of the urban area, respectively.

After the main structuration of the central Apennine thrust and fold belt, crustal extension has affected the Roman area since late Messinian, due to the development of the Tyrrhenian back-arc basin [47, 48]. From the Pliocene (Zanclean) to Early Pleistocene (Calabrian), neo-autochthonous marine sedimentation occurred [49, 50]. Since Early Pleistocene, regional uplift has determined the progressive shifting to a continental sedimentation, provided by a primitive drainage network controlled by the Paleo-Tiber River. Since Middle Pleistocene the interplay between glacio-eustatic sea-level variations, tectonic processes, sedimentation and volcanic activity has built the geological framework of the Roman area ([51], and references therein). During periods of sea-level, fall erosion occurred, while deposition took place during phases of sea-level rise, filling previously excavated incisions. A thick succession of pyroclastic-flow deposits, from both the Colli Albani and Monti Sabatini, and subordinated Sabatinian air-fall deposits, interfingers with the continental sediments. After the last volcanic eruptions 36 ka [52], the volcanic plateau was deeply incised during the Last Glacial, also as a consequence of the intervening regional uplift [38, 53]. Eventually, the paleovalleys were filled by fluvial deposits as a consequence of the sea-level rise after the last glacial termination [54].

## Results

### Reconstructed geologic sections

#### Saccopastore (41°56' 07"N; 12° 31' 39"E)

**Fig 3:**
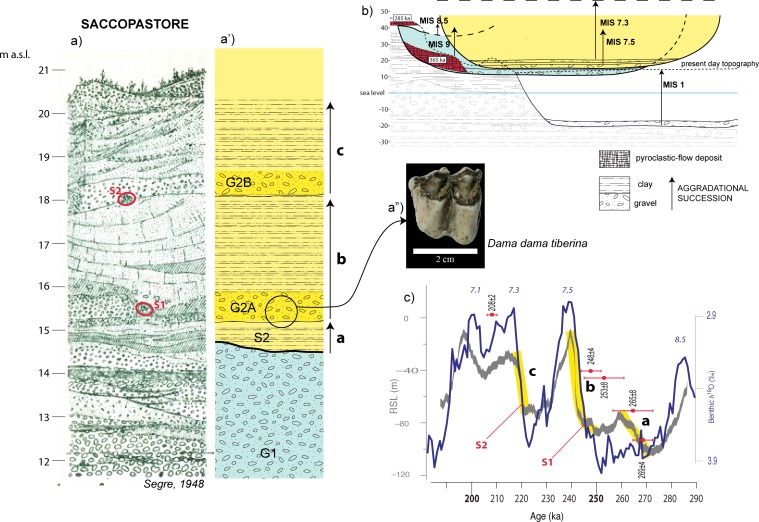
a: Stratigraphy of Saccopastore by [5]) reporting the position of the two Neanderthal skulls (S1 and S2). a': interpreted stratigraphy (modified by [3]), showing the occurrence of three aggradational phases (a, b, c) erosionally above the basal gravel layer of the Aurelia formation (G1). a": upper molar of *Dama dama tiberina* determined in the present work through examination of the fossil remains now located at the " Museo Nazionale Preistorico Etnografico Luigi Pigorini" in Rome, Italy (photo by kind permission of F. Rubat Borel, Director of Museo Pigorini). b: chronostratigraphic constraints providing correlation with the Marine Isotope Stages (MIS). c: curve of global relative sea level (RSL) in the time span 290–200 ka [56], showing the occurrence of three consecutive peaks (a, b, c, highlighted by the yellow bars) at around 270 ka, 245 ka, and 220 ka. The red dots are the ^40^Ar/^39^Ar ages (in ka) of interbedded volcanic layers constraining phases of aggradation of the Vitinia Formation at the type-locations of Pantano di Grano and Vitinia [35], and in Torre in Pietra [57]. The Oxygen isotope curve for the same time span is also reported [44]. The three aggradational successions recognized in Saccopastore (a, b, c) are correlated with the three sea-level rises occurring during MIS 7, providing indirect ages of 245 ka and 220 ka for the two skulls, S1 and S2, respectively.

The stratigraphic section of Saccopastore, described in detail by [5] (Fig 3A), was correlated by [3] with the aggradational successions of MIS 9 and MIS 7, corresponding to the local Aurelia and Vitinia Formations [7], respectively, thanks to the geochronologic and geometric constraints shown in Fig 2B. Moreover, these authors recognized the occurrence of three aggradational sub-cycles (a, b, c, Fig 3A') above the basal gravel layer of the Aurelia Formation (G2 in Fig 3A') containing a skull of “archaic” *Elephas antiquus* (recte *Palaeoloxodon antiquus*) [2, 4, 5, 6]. In the present study, we have analyzed several fossil specimens recovered in these upper layers at Saccopastore during the early investigation at this site [1, 5 and references therein], presently held at the "Pigorini" Museum in Rome. The following taxa have been identified:

Panthera spelaea, Hippopotamus amphibius, Bos primigenius, Dama dama tiberina.

Identification of the latter taxon, a less evolved subspecies of modern fallow deer, is provided by an upper molar (Fig 3A") showing robust enamel, clearly little developed entostyle, a discontinuous anterior cingulum, and a barely discernible posterior cingulum. These features, along with the tooth size (length: 23.1 mm; breadth: 22.7 mm) allow it to be recorded as *D*. *dama tiberina*, differentiating it from the upper molars of both *D*. *clactoniana* and *D*. *dama dama* (see [55]).

Notably, *D*. *dama tiberina* is a marker of the Vitinia FU [8, 9], based on its occurrence encompassing MIS 8.5 through MIS 7, as opposed to *D*. *dama dama*, replacing it in MIS 5, and *D*. *clactoniana*, disappearing after MIS 9.

The occurrence of *Dama dama* sp., as reported by [5], is limited to the lowest gravel layer G2A and the underlying sandy clay layer S2 of the Vitinia Formation (Fig 2A'). Based on elevation around 15 m a.s.l. of these layers (Fig 3A and 3A'), attribution to the MIS 8.5 Via Mascagni succession [13] is excluded (see Fig 3B), restricting correlation of the upper sedimentary deposits of Saccopastore to MIS 7. Moreover, an early aggradational phase lacking a coarse-sized basal deposit (a in Fig 3A'), is recognized below two aggradational cycles with gravel at the base (b and c in Fig 3A'). Such a feature is observed also at the type-section of the Vitinia Formation in Pantano di Grano [35], where interbedded volcanoclastic layers provide geochronologic constraints, correlating these three depositional cycles with as many consecutive sea-level jumps evidenced in the relative sea-level curve [56] in the time span 270–220 ka (a, b, c, in Fig 3C). Extending this correlation to the three aggradational phases in Saccopastore, allows us to provide distinct ages of 245 ka and 220 ka for the two skulls S1 and S2 (Fig 3C).

#### Via Mascagni/Tangenziale Est (41° 55' 17"N; 12° 30' 46"E)—Sedia del Diavolo (41° 55' 46"N; 12° 31' 22"E)

**Fig 4:**
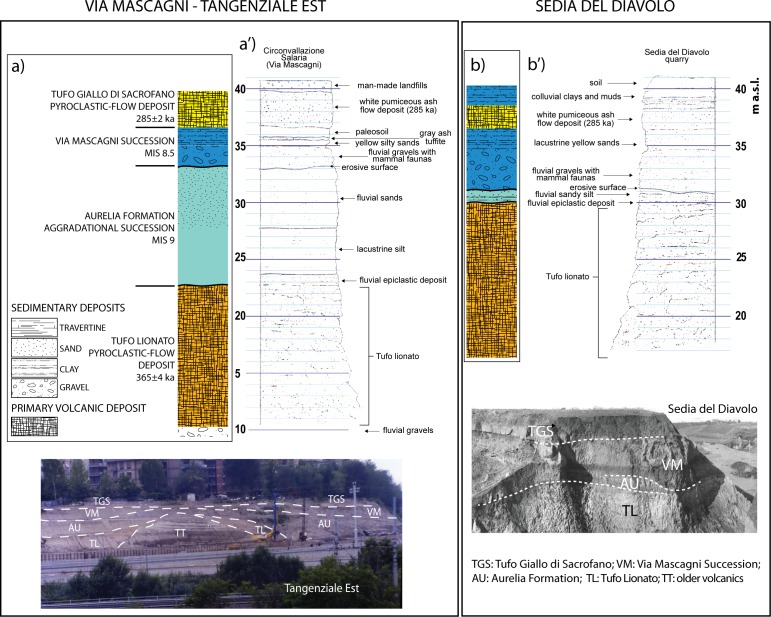
a-b: stratigraphic columns of Via Mascagni/Tangenziale Est and Sedia del Diavolo geologic sections, interpreted from stratigraphic schemes by [60], re-drafted here (a' and b') for illustrative purposes only. Original outcrop photographs by F. Marra.

The stratigraphic setting reconstructed in the composite section of Via Mascagni/Tangenziale Est (Fig 4A) was directly investigated by [58] during the excavations performed to realize the tract named "Circonvallazione Salaria" of the Tangenziale Est urban motorway. The entire pack of sedimentary deposits exposed in this area was attributed to the Aurelia Formation (MIS 9) based on their stratigraphic position above the pyroclastic-flow deposit of Tufo Lionato [58], whose age at that time was assessed at 0.338 Ma [59].

Successively, the pyroclastic-flow deposit on top of the sedimentary deposits, sampled at an outcrop in Via Cheren, 200 m south of Sedia del Diavolo, was dated 285±2 ka [30] (this age and all the following ones reported in the text are re-calculated according to the Alder Creek sanidine standard calibration at 1.194 Ma [61], therefore they may differ slightly with respect to the original published data), allowing [62] to correlate the uppermost portion of the sedimentary deposits, including the gravel layer and the overlying sandy-clayey horizons, with the minor sea-level oscillation associated with MIS sub-stage 8.5.

A detailed stratigraphic column of Circonvallazione Salaria was reported by [60] (Fig 4A') to compare the stratigraphy of the no longer exposed section of Sedia del Diavolo described in [18] (Fig 4B'). Despite being aware of the age of the Tufo Giallo di Sacrofano (TGS, from now onward), these authors have attributed the entire succession to MIS 9 and argued that the first appearance of *Equus hydruntinus* and *D*. *dama tiberina*, both occurring in the deposits of Sedia del Diavolo, could not be considered as a distinctive feature of the Vitinia FU [8, 9], which is approximately correlated with the homonymous Vitinia Formation and MIS 7. In contrast, the Torre in Pietra FU is approximately correlated with the Aurelia Formation and MIS 9. However, [13] have remarked that the remains of *E*. *hydruntinus* and *D*. *dama tiberina* occur within the deposit of the Via Mascagni succession, which is correlated with the later sub-stage 8.5. These taxa have never been found in the deposits of MIS 9 in Italy, justifying the introduction of a distinct Vitinia FU which encompasses MIS 8.5 and MIS 7, as opposed to the Torre in Pietra FU, limited to MIS 9.

We have re-interpreted the stratigraphic sections drawn by [60] according to the geochronologic constraints provided by TGS and Tufo Lionato (TL, from now onward) pyroclastic flow, according to the re-calculated ages of 285±2 ka and 365±4 ka respectively [63, 64], and we have condensed them in the stratigraphic columns of Fig 4A and 4B.

#### Monte delle Gioie (41° 56' 45"N; 12° 30' 44"E)

**Fig 5:**
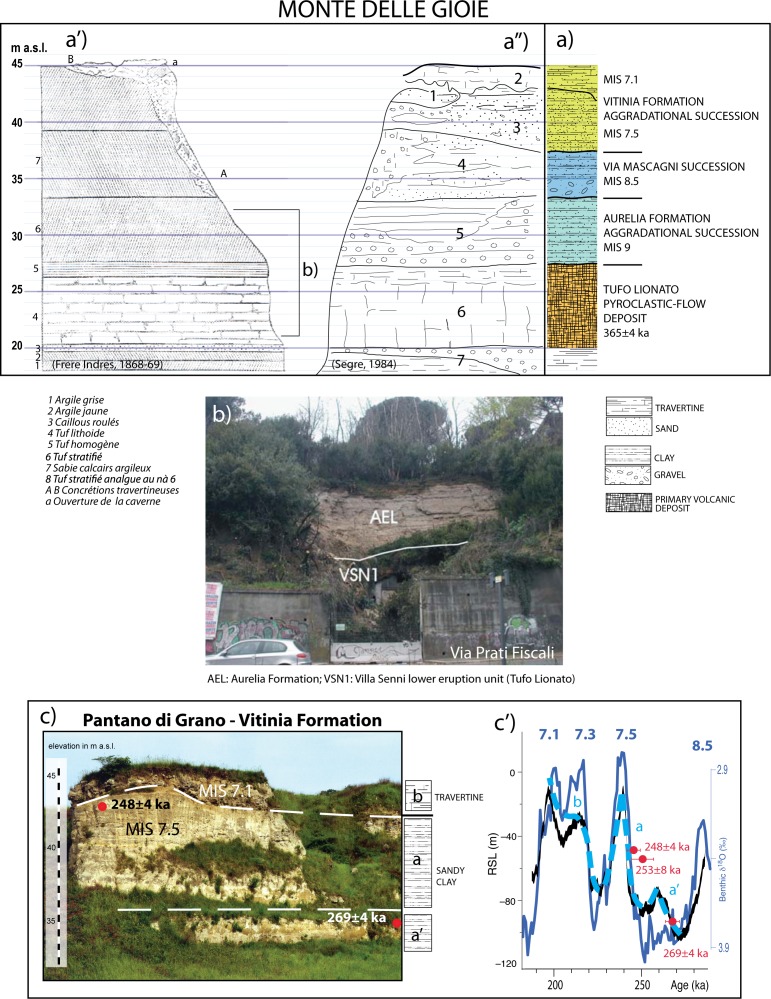
a: Stratigraphic column of Monte delle Gioie geologic section interpreted from stratigraphic schemes by [65] (a') and [6] (a"), re-drafted here for illustrative purposes only. b: Photograph of the outcrop in Via Prati Fiscali [66] (reproduction authorized by SIGEA, S1 File) showing the sedimentary deposit of Aurelia Formation (AEL) overlying the Tufo Lionato pyroclastic-flow deposit (VSN1). c: Outcrop of the Vitinia Formation deposit in Pantano di Grano (Ponte Galeria area, southwest of Rome. Photograph taken by F.M.) showing stratigraphic position of the sample dated 269±4 ka [35] providing correlation with an early phase of sea-level rise (a' in Fig c') at the onset of MIS 7 (Oxygen isotope record by [44]), evidenced by the Relative Sea Level curve by [56]. c': Full aggradation of the Vitinia Formation during major sea-level rise associated with MIS 7.5 (a) is constrained by age of 253±8 ka of a volcanic layer interbedded with the sedimentary deposits at the type-section of Vitinia [7]. A third aggradational cycle (b), correlated with the sea-level rises during MIS 7.3–7.1 is evidenced by the occurrence of travertine deposits unconformably overlying the clayey section in Pantano di Grano (c).

The stratigraphy of Monte delle Gioie hill (Fig 5) was first described by [65], and was correlated to that of Sedia del Diavolo by [4, 5, 6], who recognized also the similarity of the lithic industries recovered within the gravel layer occurring approximately at the same elevation (ca. 33 m a.s.l., see Fig 5A) in both sections. Later, [18] proposed the term "proto-Pontinian" for these lithic industries because of their intermediate features, in contrast to the typical lower Palaeolithic Acheaulian industries of this region and the local Mousterian facies, referred to as "Pontinian".

A detailed description of the Monte delle Gioie stratigraphy was reported in [6]). However, based on field investigation performed for the present study at the outcrops still exposed in Via Prati Fiscali (Fig 5B), we have verified that the several vertical and lateral heterogeneities reported in [6] (Fig 5A") with respect to the homogeneous composition of the layers described in [65] (Fig 5A'), are unsupported by field evidence. Indeed, the layer overlying the TL pyroclastic-flow deposit (AEL and VSN1 in Fig 5B, respectively) is constituted by well-bedded, centimeter-thick clay layers with fresh water gastropoda, remarkably similar to the analogous lacustrine clay deposit of the Aurelia Formation occurring at the outcrop of Tangenziale Est. In the stratigraphic column of Fig 5A we have re-interpreted the stratigraphic section drawn by [65] and by [6], based on chronostatigraphic constraints achieved through correlation with the Via Mascagni and Sedia del Diavolo sections of Fig 4. We have confidently attributed the lower portion of the sedimentary deposits above TL to the Aurelia Formation and to the Via Mascagni succession, based on their straightforward lithostratigraphic correlation with Via Mascagni and Sedia del Diavolo sections. We have interpreted the upper portion of the section, including two layers separated by an unconformable surface reported by [6] (2 and 3 in Fig 5A"), as a third depositional cycle overlying that of Via Mascagni succession, according to its geometric (absolute elevation) and lithostratigraphic features, similar to those of the Vitinia Formation observed in other outcrops of this region. In particular, there is a remarkable similarity with the deposit of the Vitinia Formation investigated and geochronologically constrained within MIS 7 occurring at Pantano di Grano section, southwest of Rome [35] (Fig 5C and 5C'). Fig 5C' shows that an early aggradation of the Vitinia Formation since 269±4 ka is evidenced by the age of a volcanic layer in Pantano di Grano (Fig 5C), whereas a full aggradation was completed by 253±8 ka during MIS 7.5 (Fig 5C'), as evidenced by the age of another primary layer sampled at the type-section in Vitinia [7], as well as by a reworked volcanic layer occurring at higher elevation in Pantano di Grano, yielding age ≥248±4 ka (Fig 5C and 5C'). Moreover, the occurrence of a travertine deposit unconformably overlying the major aggradational succession in Pantano di Grano (Fig 5C), testifies the occurrence of a later aggradational cycle correlated with a new sea-level oscillation. Elevation of this deposit 43–45 m a.s.l. rules out correlation with the later glacio-eustatic cycle of MIS 5, whose fluvial terrace occurs 38 m a.s.l. in this area [3], constraining its deposition during the isotopic peaks 7.3–7.1.

#### Ponte Mammolo (41° 55' 25"N; 12° 30' 00"E)

**Fig 6:**
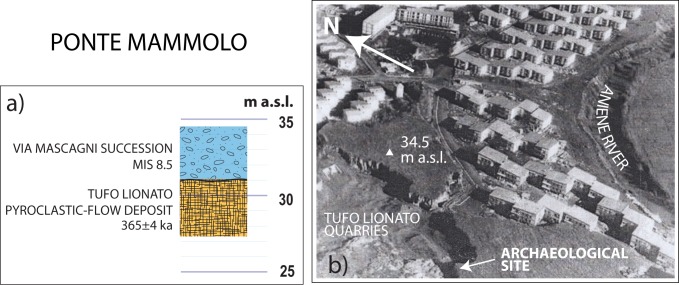
a: Stratigraphic column of Ponte Mammolo geologic section, based on description in [16] and on photograph of the original quarry excavations (b; www.archidiap.com; under Creative Commons Attribution License), showing the Tufo Lionato (TL) escarpment to the top of which the gravel and sand layer with the lithic industry and a human femur were recovered.

No geologic section showing the stratigraphic context in which the lithic industries and the human femur recovered at Ponte Mammolo occurred exists in the relevant literature. However, a description of the lithological features of the deposit and its stratigraphic relationship with respect to the Tufo Lionato pyroclastic-flow deposit (TL), extensively outcropping at that location, was provided in [16]. These authors report (p. 1–3) that <<All the remains (femur included)… …come from the upper part of the terrace, which is above the right bank of the Aniene…which overhangs the "lithoid tufa" (i.e.: TL)… …It consists of gravel and sand layers.>> An aerial photograph of this area taken in the year 1974 (Fig 6B) shows the geomorphologic features at the quarries where the archaeological find was made and, combined with description in [16], allowed us to position the site upon the modern morphology (Fig 6B). During our field survey in Ponte Mammolo we verified that the top of the terraced surface on the right bank of the Aniene River culminates at 34.5 m a.s.l., and that the steep scarp formed by the lithified TL is exposed up to 30.5 m a.s.l. (Fig 6B), constraining elevation of the gravel and sand layers described by [16]) between these values, and providing stratigraphic correlation with the Via Mascagni succession cropping out at the other sites of the Aniene Valley.

#### Tor Cervara (41° 55' 40"N; 12° 34' 52"E)

**Fig 7:**
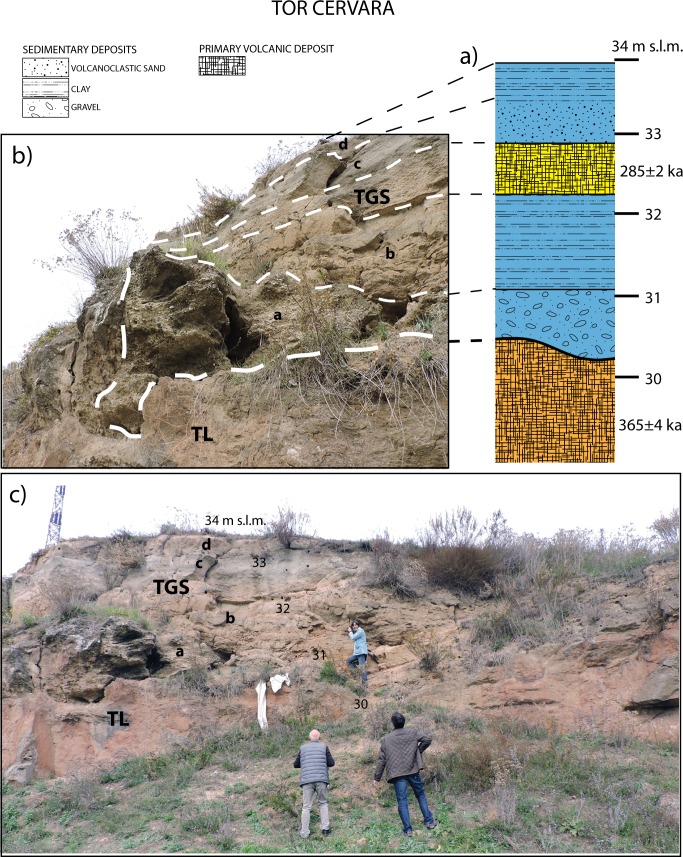
a: Representative stratigraphic column of the deposit correlated with the Via Mascagni succession based on (b-c) identification of the Tufo Giallo di Sacrofano (TGS; 285±2 ka [63]) pumice-flow deposit within the sedimentary deposit with gravel (a) at the base, erosionally overlying the Tufo Lionato pyroclastic-flow deposit (TL; 365±4 ka [64]). Photographs taken by F.M., the persons appearing in inset c are F.M., C.P, and M.F.R.

We have investigated the geologic setting of Tor Cervara locality where a stratigraphy very similar to that described by [16] at the site of Ponte Mammolo, located 1 km southwest, is exposed (Fig 7). A flat surface, whose culmination is reported at 34 m a.s.l. on the 1:25.000 topographic map by Istituto Geografico Militare, occurs on the left bank of the Aniene River (Fig 7C). Exposure along a quarry cut into the TL shows a sedimentary succession with a discontinuous sandy gravel layer at the base, erosionally above the volcanic deposit (Fig 7B and 7C). The gravel (layer a in Fig 7B) reaches up to 1m in thickness within erosive sags on top of the eroded surface of TL and is followed by ca. 2 m of silty clay, fluvial-lacustrine deposits (layer b). Above it, a ca. 50 cm thick pumice-flow deposit (TGS) occurs, followed by a volcaniclastic sand layer (c) and more silty clay (layer d).

The representative stratigraphic column of Tor Cervara (Fig 7A) has been used to constrain further geometry of the Via Mascagni aggradational succession along the Aniene Valley.

#### Casal de' Pazzi (41° 55' 42"N; 12° 33' 51"E)

**Fig 8:**
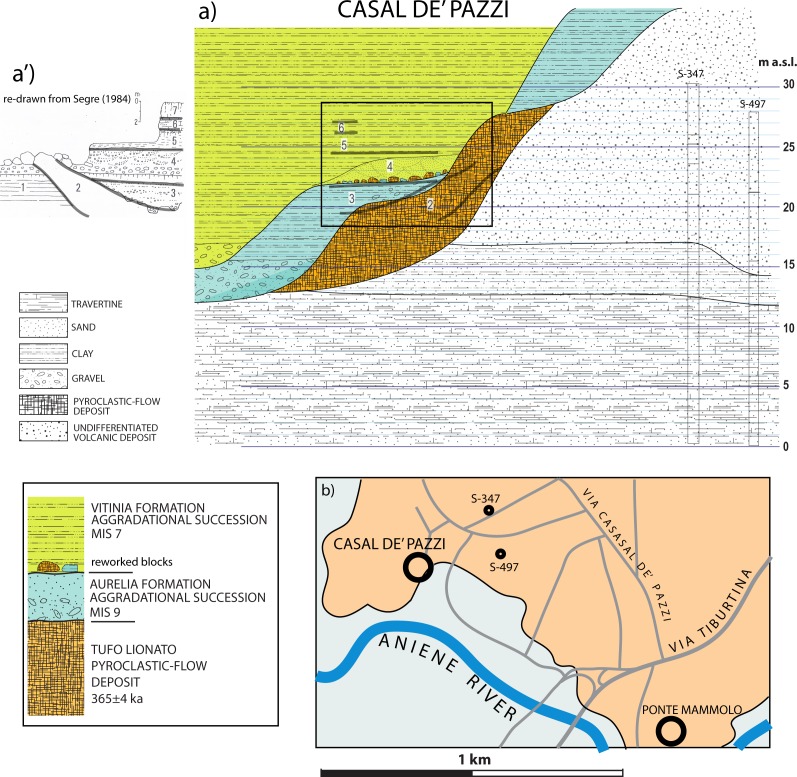
a) Stratigraphic section of Casal de' Pazzi reconstructed, based on re-interpretation of stratigraphic scheme (a') by [6], re-drafted here for illustrative purpose only, and on sub-surface geologic setting described in boreholes drilled near the archaeological site for civil engineering purpose, stored in the databank by Istituto Nazionale di Geofisica e Vulcanologia (S-347, S-497; Supplementary material S2 File). b: Location map.

The stratigraphic section of Casal de' Pazzi reported by [6] (Fig 8A') shows two fining-upward deposits (layers #3 and #4 in Fig 8A') erosionally above the Tufo Lionato pyroclastic-flow deposit (TL) (#2 in Fig 8A'), separated by a paleosoil. The lowest succession is described as fluvial silt with lens of gravel at the base, ca. 2 m thick. Above the paleosoil on top of this lowest succession is a conglomeratic deposit made of large, rounded blocks of TL and blocks of clayey sediment. Puzzlingly, [6] interprets the sedimentary blocks within the conglomerate as deriving from a lacustrine marl layer underlying the TL, which he reports at the base of the stratigraphic section (layer #1 in Fig 8A'). In contrast to this interpretation, direct observation made at the site of Casal de' Pazzi, combined with borehole data providing information on the stratigraphy of this area (see location in Fig 8B), allowed us to identify the sedimentary blocks as deriving from erosion of the aggradational succession of Aurelia Formation, which overlies the TL throughout the Aniene Valley [58]. Indeed, the sedimentary successions older than the TL in Rome are generally represented by massive sand and travertine deposits, alternating with diatomitic levels (Paleo- Tiber 3 deposits [33, 58]. The Tufo Lionato emplaced 365±4 ka during a low-stand of the sea-level, filling deeply incised valleys, as in the reconstructed stratigraphic scheme of Fig 8A. Therefore, it is unlikely that subsequent erosional cycles along the same valleys could affect the older sedimentary deposits, underlying the TL, and that eroded blocks of these sediments can occur within the basal horizon of the later aggradational successions.

**Fig 9:**
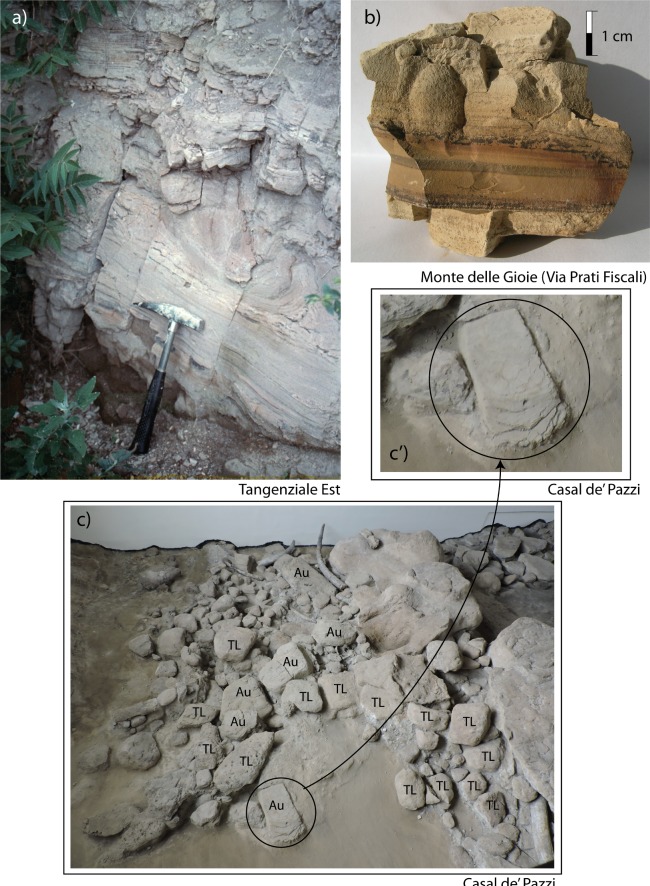
Photographs (by F. Marra) showing the characteristic texture of the sedimentary deposits of the Aurelia Formation (AU) occurring at the outcrops of Tangenziale Est (a) and Monte delle Gioie (b), compared to that of the sedimentary blocks occurring along with those of Tufo Lionato (TL) at the base of the Vitinia Formation aggradational succession in Casal de’ Pazzi (c-c’). Photographs taken at Casal de’ Pazi Museum by kind permission of Patrizia Gioia, Director.

According to this hypothesis, the stratigraphic logs of the two boreholes drilled in Via Casal de' Pazzi (S-347, S-497; original logs in S2 File) show that the top of the older sedimentary deposits occurs around 15 m a.s.l., well below the elevation at which the reworked sedimentary blocks occur, and that these deposits are essentially massive sand and travertine layers. In contrast, the fluvial-lacustrine deposits of the Aurelia Formation overlying the TL are characteristically constituted by alternating very fine layers of white carbonatic muds and brown clay, displaying a typical varved texture that is observed at all the outcrops of the Aniene Valley, like those no longer exposed at Tangenziale Est and those exposed at Monte delle Gioie (Fig 9). We have verified that the large blocks occurring at the base of the sedimentary deposit of Casal d’ Pazzi, along with those of TL, show this peculiar texture (Fig 9), recognizing their provenance from the eroded portion of the Aurelia Formation that overlies the TL deposit along the investigated sector of the Aniene Valley. This fact implies an age younger than MIS 9. Moreover, we exclude correlation with the Via Mascagni succession, based on the elevation around 25 m a.s.l. for the deposit: much lower than the gravel layer representing the base level of the succession correlated with MIS 8.5 (see Figs 3, 5 and 6).

We have therefore re-interpreted the stratigraphic section of [6] and we have reconstructed the stratigraphic setting at Casal de’ Pazzi, merging direct observations and borehole data in Fig 8A. The conglomeratic deposit in which the human parietal, the lithic industries and the vertebrate fossil remains were recovered [11], is constituted by large fallen blocks from a nearby escarpment of Tufo Lionato overlain by deposits of the Aurelia Formation, which was originated by erosion during the sea-level fall of MIS 8. The fluvial terrace at the base of the escarpment very likely hosted a temporary meander, and the blocks were partially re-elaborated by action of the flowing water during the regressive phase of MIS 8. Several lithic artefacts recovered in this horizon, such as a bifacial tool and a chopper [11], typically occurring in the Acheulean assemblages of this region [67]), were likely embedded within the deposits of the Aurelia Formation and were reworked during this erosive phase. Similarly, the faunal assemblage recovered from this horizon displayed different degrees of fluitation and abrasion [11]. More clastic material began to accumulate rapidly within the river incision, along with the less abraded lithic industries and vertebrate remains, during the period following glacial termination at the onset of MIS 7, as a consequence of the rise in sea-level, which eventually led to the filling of the fluvial valley by the fine-grained portion of the Vitinia Formation aggradational succession. The cranial fragment recovered at Casal de' Pazzi does not show a high degree of abrasion [15] and should be considered to be emplaced during this later aggradational phase.

## Discussion

### Chronostratigraphic setting

**Fig 10:**
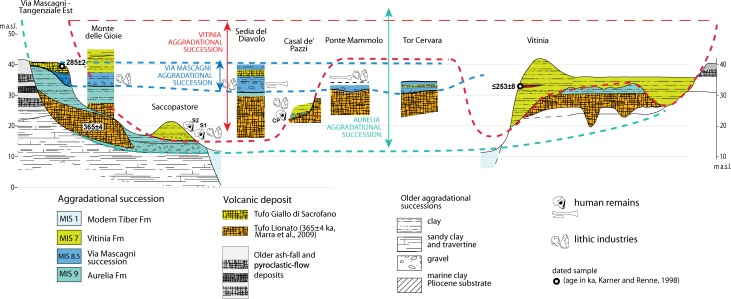
Stratigraphic columns of the sites of the Aniene River Valley yielding hominin remains and lithic industries are projected along a transect (see inset b for location) along with other reference sections. Geochronologic and geomorphologic constraints providing correlation with the aggradational successions deposited in response to sea-level rise during glacial terminations are shown (see text for details).

The five geologic sections investigated in the present study (Sedia del Diavolo, Monte delle Gioie, Ponte Mammolo, Tor Cervara, Casal de' Pazzi) are ideally projected on a longitudinal transect along the Aniene Valley (Fig 10), which includes the composite section of Via Mascagni/Tangenziale Est and the type-section of the Vitinia Formation [7], in which direct geochronologic constraints were achieved and used to provide correlation with MIS 7 for the aggradational succession of Saccopastore [3]. These constraints serve as reference sections for the other sites of the Aniene Valley where hominin remains were found.

**Fig 11:**
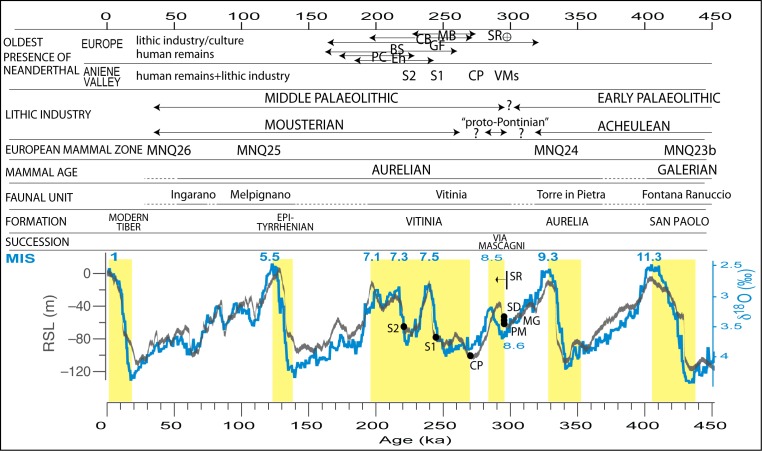
Ages provided in this study of the hominin remains of the Aniene Valley (black dots) through correlation of the hosting aggradational successions with the glacio-eustatic sea-level rises (RSL curve by [56]) are compared to the oldest evidence of Neanderthal presence in Europe ([80], and references therein). Yellow boxes represent the aggradational phases in the near-coastal area of Rome, constrained by ^40^Ar/^39^Ar ages on intercalated volcanic layers (see Fig 2) providing their correlation with the RLS curve and the Marine Isotopic Stages (MIS). Oxygen isotope curve by [44].Legend: MB: Maastricht-Belvedere, unit IV, 250±20 ka [28]; GF: Grotte Vaufrey, level X, 246±76 ka [27]; CB: Cova de Bolomor, level XIV a&b, 233±35 ka [24]; Eh: Ehringsdorf, ca. 230 [81], 186–242 ka [82]; PC: Pontnewydd cave, 200±25 ka [83]; BS: Biache-Saint-Vaast, IIA, 175±13 ka, 253 +53/-37 ka [21]; SR: Solent River [23]. VMs: Via Mascagni succession sites (Sedia del Diavolo (SD), Ponte Mammolo (PM), Monte delle Gioie (MG)); CP: Casal de’ Pazzi; S1 Saccopastore 1; S2: Saccopastore 2.

An upper aggradational succession is recognized at four of these sections and correlated with the Via Mascagni succession, deposited during MIS 8.5 [13]. This is characterized by a basal gravel layer ranging 1–4 m in thickness and occurring erosionally above fluvial-lacustrine clay of the Aurelia Formation or the pyroclastic-flow deposit of TL at approximately the same elevation in all sections (31–33 m a.s.l., Fig 10). The gravel is covered by an approximately 2-meter-thick sandy clay fluvial-lacustrine deposit. The occurrence of the Pyroclastic-flow deposit of TGS (285±2 ka [63]) on top of this aggradational succession has been reported in Via Mascagni and at Sedia del Diavolo [4, 58, 60] at elevation ranging 37–40 m a.s.l. (Fig 4). Age of 285±2 ka for the TGS allowed [33] correlation of the underlying fining-upward succession with the sea-level rise during sub-stage 8.5 (Fig 11) and its definition as a minor aggradational succession (Via Mascagni succession), emplaced shortly after that of the Aurelia Formation, which in turn is correlated with MIS 9 based on age of 365±4 ka [64] of the underlying TL pyroclastic-flow deposit (Figs 10A and 11). Elevation, thickness and lithostratigraphic features of the exposed deposits allow for a straightforward correlation of Monte delle Gioie section [6] (Fig 5) with the near Via Mascagni and Sedia del Diavolo sections (Fig 10A). The deposits of the Via Mascagni succession are no longer visible at Ponte Mammolo, where we have identified them based on the description made by [16] of the layers from which the human remain attributed to a proto-Neanderthal type was recovered: a gravel and sand horizon occurring on top of the terraced surface formed by the TL pyroclastic-flow deposit (Fig 6). Elevation of this surface between 30 and 34.5 m a.s.l. provides stratigraphic correlation with the Via Mascagni succession cropping out at the other sites of the Aniene Valley (Fig 10). This correlation is constrained further by stratigraphy at the site of Tor Cervara (Fig 7), where we have observed the TGS overlying a 2 meter-thick sandy clay deposit above a discontinuous, up to 1 meter- thick gravel layer occurring within erosive sags on top of the eroded surface of TL, approximately 31 m a.s.l.. At Casal de' Pazzi, stratigraphic section by [6] (Fig 8) shows two fining-upward deposits erosionally above the TL, separated by a paleosoil. The lowest succession is described as fluvial silt with lens of gravel at the base, ca. 2 m thick. Above the paleosoil on top of this lowest succession is a conglomeratic deposit made of large, rounded blocks of TL and blocks of clayey sediment, in which the parietal attributed to an archaic *Homo* was recovered along with the lithic artefacts and vertebrate fossils [11]. Direct observation made at the site of Casal de' Pazzi, combined with borehole data providing information on the stratigraphy of this area (Fig 8), allowed us to identify the sedimentary blocks as deriving from erosion of the aggradational succession of Aurelia Formation, which overlies the TL throughout the Aniene Valley (Fig 10A). This fact implies an age younger than MIS 9. Moreover, we exclude correlation with the Via Mascagni succession, based on the elevation around 23 m a.s.l. for the conglomeratic deposit at Casal de Pazzi: much lower than the gravel layer representing the base level of the succession correlated with MIS 8.5 (Fig 10). Therefore, we correlate the deposit of Casal de' Pazzi and the human remain hosted in it with the initial stages of aggradation of the Vitinia Formation around 270 ka [7, 35] (Fig 2). Post-glacial sea-level rise during MIS 7 is indeed characterized by several pulses (see relative sea-level curve in Fig 2) to which an equivalent number of aggradational phases should correspond, as discussed in [3]. Accordingly, these authors interpreted the presence of two fining-upward successions with gravel at the base in Saccopastore as reflecting the occurrence of two strong deglaciation phases, originating the consecutive sea-level jumps at the onset of MIS 7.5 and 7.3. Therefore, in Fig 2 we attribute the correspondent ages of 245 and 220 ka to the two skulls (S1 and S2) recovered in these two gravel layers, refining the previously undistinguished ~250 ka age. We estimate a slightly older age of+ ~270 ka for the human parietal of Casal de' Pazzi, consistent with evidence for a still incomplete glacial termination provided by its position directly above the erosive surface at the base of the deposits of the Vitinia Formation (Fig 8). Finally, we have assessed a tightly constrained age of 295 ka (i.e.: between the onset of sea-level rise associated with MIS 8.5 and emplacement of TGS at 285 ka, Fig 2) for the human remains and the associated lithic industries of Sedia del Diavolo and Ponte Mammolo, and for the lithic industry of Monte delle Gioie, occurring within the deposits of the Via Mascagni succession.

### Paleonthological remarks

The vertebrate fauna associated with the human remains and the lithic industries at all the locations investigated is quite homogeneous and is characterized by a few species of carnivores (e.g., the spotted hyena *Crocuta crocuta*, the wolf *Canis lupus*) and several herbivores (e.g., the steppe rhino *Stephanorhinus hemitoechus*, the straight-tusked elephant *Palaeoloxodon antiquus*, the auroch *Bos primigenius*, the wild horse *Equus ferus*, less evolved subspecies of red deer, *Cervus elaphus* ssp.), with the discriminating presence of the European wild ass *Equus hydruntinus*, and an archaic form of modern fallow deer, *Dama dama tiberina* [8, 9] (Table 1), both appearing for the first time in Italy during MIS 8.5 [13]. In particular, *D*. *dama tiberina* is marker of the Vitinia FU [8, 9]).

**Table 1:** Mammals from the sites of the Aniene Valley referred to the Vitinia Faunal Unit. Data from [3, 9, 16].

Locations	Saccopastore	Casal de’ Pazzi	Ponte Mammolo	Sedia del Diavolo	Monte delle Gioie
Land Mammal Age	Aur	Aur	Aur	Aur	Aur
Faunal Unit	Vit	Vit	Vit	Vit	Vit
MIS	7	7	8.5	8.5	8.5
*Canis lupus*		X	X	X	
*Ursus spelaeus*			X		
*Meles meles*				X	
*Panthera spelaea*	X				
*Crocuta crocuta*		X			
*Palaeoloxodon antiquus*	X	X		X	X
*Stephanorhinus* sp.	X	X		X	
*Stephanorhinus hemitoechus*				X	X
*Equus ferus*	X	X	X	X	
*Equus hydruntinus*	X			X	
*Hippopotamus* sp.	X				
*Hippopotamus* cf. *amphibius*		X		X	X
*Sus scrofa*		X		X	
*Megaloceros giganteus*			X		
*Cervus elaphus* ssp.	X	X		X	X
*Dama dama tiberina*	X			X	X
*Dama dama* ssp.		X			
*Capreolus capreolus*		X			
*Bos primigenius*	X	X	X	X	X

### Remarks on lithic assemblages

All the lithic industries recovered at the investigated sites of the Aniene Valley were described at the time of their discovery using terminology and classification criteria that nowadays are in part superseded, and no further study of the materials according to modern methodologies (e.g.: [68]) has been conducted since the preliminary reports. The lithic industries recovered at Sedia del Diavolo and Monte delle Gioie (including 25 and 110 artefacts, respectively) were regarded as coming from the same lithic assemblage which, due to the intermediate feature between a late Acheulean and a Mousterian assemblage, was called "proto-Pontinian" [18], in order to distinguish it from the younger local Mousterian complex that has been frequently referred to as "Pontinian" (e.g. [4]). Similarly, the scanty lithic industry coming from the coeval horizon at Ponte Mammolo (ca. 20 pieces) has been described as pre-Mousterian by the authors [16]. Based on preliminary investigation (421 out of ca. 1700 pieces), the abundant lithic industry recovered in Casal de' Pazzi has also been reported as displaying similar features to those from Sedia del Diavolo and Monte delle Gioie; in particular it has been remarked [69] that the artefacts have technical and morphological characteristics which place them in intermediate position between the level m and level d from Torre in Pietra, attributed to the Acheulean and to the Mousterian respectively [70]. In contrast with the interpretation of the Casal de' Pazzi lithic industries as being part of a homogeneous, early Mousterian assemblage [69], there were previous reports of the presence of several artefacts suggestive of older, Acheulean techno-complex, such as a bifacial tool and a chopper [11]. It can be remarked that the presence of such supposedly older artefacts is consistent with the occurrence of reworked sedimentary material from the Aurelia Formation (ca. 355–325 ka) within the Vitinia Formation deposits (Figs 8 and 9).

A re-evaluation of the typological characteristics of the artefacts and their classification is beyond the scopes of the present work, which is aimed at providing precise age to the sedimentary deposits in which they were recovered. However, the strict geochronologic constraints provided here, which indicate an age of 295 ka for the assemblages recovered at the coeval sites of Sedia del Diavolo and Monte delle Gioie, and a slightly younger but well constrained age of ~270 ka for the rich assemblage recovered at Casal de' Pazzi, imposes a re-examination of the materials, in the light of the considerable advances made by studies on the Lower-Middle Paleolithic transition in recent years (e.g.: [40, 71, 72]), and based on a more up-to-date methodological approach.

It can also be noted that, since the studies performed in the 60's [18] and since the preliminary reports published in 1984–90 [11, 69], no complete study has ever been conducted on these important lithic assemblages, falling within a crucial time interval leading to the origin of the Mousterian in Italy, characterized by the appearance of the Levallois core reduction technology (Mode 3) during the Middle Paleolithic transition [73, 74]). This occurrence has been shown to be broadly coeval in Europe and pinpointed at the MIS 9–8 transition [22, 24, 25, 27, 28], or to the late MIS 9 [23]. However, it has been claimed that early occurrences of Levellois technology have occurred since MIS 11/10 transition at the Gualdo San Nicola site of central Italy [75]. In contrast, at the eastern door of Europe the first appearance of Levellois has been geochronologically constrained at 335–325 ka at Nor Geghi 1, Armenia [76].

Based on data presented in this paper, the lithic assemblages of the Aniene Valley characterized by transitional features between the Acheulian and Mousterian local cultures are dated at 295 ka during MIS 8.5 (Fig 11). Remarkably, using a very similar approach based on correlation of fluvial terrace incisions of the Solent River (England) with the Oxygen Isotope Stages timescale, [23] it has been found from their modeling that there is a better fit with the various data than if Levallois had first appeared in MIS 9b. This isotopic event corresponds to MIS 8.6 in the nomenclature of [77] adopted in this paper, in very good agreement with data from the Aniene Valley recording the possible local equivalent of this occurrence during the following sub-stage 8.5 (Fig 11). Similarly, the early appearance in Italy of the Levallois reduction concept has been recently suggested to occur before MIS 8, based on the re-evaluation of the lithic assemblage of Cave dall'Olio (Bologna, northern Italy) [26, 78]. Data from the Aniene Valley confirm that the spread of the Levallois method in Europe was a fast process, since it is recorded at the same time in different places in England, Spain, France, and across Italy. Based on the new dates produced for level d of Torre in Pietra [57] and on the model presented in this work, it is also now clear that the hypothesis of a late spread across Italy of this key reorganization of core technology, a supposition previously based on evidence from the lower units of San Bernardino Cave, from northern Italy [79], is invalid.

However, it should be remarked that a detailed and modern analyses of the lithic industries recovered at the sites of the Aniene Valley has not yet been provided, preventing the safe attribution of the materials to a specific techno-complex.

## Conclusions

According to their attributions, ranging from proto-Neanderthal (those recovered in the deposits of the Via Mascagni succession) to archaic Neanderthal (the skull from the lowest Saccopastore layer occurring in the Vitinia Formation deposits), the hominin remains associated with the lithic industries considered transitional to the Middle Palaeolithic can be ascribed to early Neanderthal populations, living in the time span 295–220 ka in the Aniene Valley (Fig 11).

Available age determinations on the Neanderthalian fossils in Europe are often contrasting, depending on the dating method applied, and are poorly constrained, with associated errors in the order of several tens of thousands of years (e.g.: [80], and references therein). The ages for the oldest direct Neanderthal evidence in Europe, either from Ehringsdorf [81, 82] or from Biache-Saint-Vaast [21], are still strongly debated, with supposed ages ranging from186–242 ka and 175–253 ka, respectively (Fig 11). At Pontnewydd cave, Neanderthal occurrence is dated 200±25 ka [83].

In contrast, our method of correlation allows us to provide well constrained ages for the human remains that have been recovered within the aggradational successions deposited in response to the sea-level rises which occurred 295, 270, 245 and 220 ka. Errors associated to these ages should be considered in the same order as those of the ^40^Ar/^39^Ar ages constraining the sedimentary deposits, ranging 2–8 ka, or those associated with calibration of the oxygen isotopes curve, in the order of ±5 ka [33]. For example, the occurrence of the Lower-Middle Paleolithic transitional lithic assemblages is strictly dated at 291±4 ka, thanks to the combined ^40^Ar/^39^Ar age of 285±2 ka yielded by the pyroclastic deposit sealing the deposition of the sedimentary successions of Monte delle Gioie and Sedia del Diavolo, combined with the age of the sea-level rise in this time span, placed between 295 and 287 on the isotopes record of [44] (Fig 11). If we use the independently constrained chronology of the relative sea-level curve by [56], this eustatic event (and therefore deposition of the sedimentary successions) is pinpointed between 298 and 292 ka (i.e.: 295±3 ka) (Fig 11). This result is in perfect agreement with the other European recording dated with the same methodological approach and the same precision at the Solent River, pinpointing the appearance of Levellois to MIS 9b [23], which on the curve by [44] falls at 295 ka and corresponds to sub-stage 8.6 (Fig 11).

With respect to recent work by [3], in the present study we extend the attribution of four more hominin remains to Neanderthal-type individuals, and establish precise ages for all of them and for the associated lithic industries. Moreover, we refine and reinforce the age previously inferred for the two skulls recovered in Saccopastore, showing that besides being among the oldest and best dated Neanderthal occurrences in Europe, all these finds testify to a long lasting and numerous frequentation of the Aniene Valley, 295 through 220 ka.

## Supporting Information

S1 File Permits. (PDF)

S2 File Stratigraphic logs of two boreholes drilled near the archaeological site of Casal de' Pazzi. (DOC)
