# Supplementary material for: The Aggradational Successions of the Aniene River Valley in Rome: Age Constraints to Early Neanderthal Presence in Europe
Source: PLoS One. 2017 Jan 26;12(1):e0170434. doi: 10.1371/journal.pone.0170434 (PMC5268786; doi:10.1371/journal.pone.0170434)
Supplement: S1 File — (PDF) [file pone.0170434.s001.pdf]

AUTHORIZATION TO STUDY THE FAUNAL REMAINS FROM SACCOPASTORE HOSTED AT MUSEO NAZIONALE PREISTORICO ETNOGRAFICO "LUIGI PIGORINI" AND TO THE REPRODUCTION OF PHOTOGRAPHS IN PLOS ONE UNDER THE SPECIFIC CREATIVE COMMONS ATTRIBUTION LICENSE (CCAL), CC BY 4.0.

Da: GABRIELLI EDITH <edith.gabrielli@beniculturali.it>
Oggetto: **Re: Istituto Nazionale di Geofisica e Vulcanologia: richiesta autorizzazione**
Data: 29 febbraio 2016 16.41.48 GMT+01.00
A: fabrizio marra <fabrizio.marra@ingv.it>
Cc: S-MNPE - DIREZIONE <s-mnpe.direzione@beniculturali.it>, RUBAT BOREL FRANCESCO <francesco.rubatborel@beniculturali.it>

Gentile dottore,
Sono certa che il dottor Rubat Borel, direttore del Museo Pigorini, che legge per conoscenza, valuterà al più presto la Sua richiesta, predisponendo nota ufficiale di risposta.
Un cordiale saluto,
Edith Gabrielli

Dott. ssa Edith Gabrielli
Direttore

Polo Museale Regionale del Lazio
Palazzo Venezia
Piazza San Marco, 49
00186 Roma

Da: RUBAT BOREL FRANCESCO <francesco.rubatborel@beniculturali.it>
Oggetto: **R: rinnovo richiesta autorizzazione**
Data: 27 aprile 2016 11.59.33 GMT+02.00
A: fabrizio marra <fabrizio.marra@ingv.it>, GABRIELLI EDITH <edith.gabrielli@beniculturali.it>, BONDIOLI LUCA <luca.bondioli@beniculturali.it>
Cc: leonardosalari@virgilio.it salari <leonardosalari@virgilio.it>, Carmelo Petronio <carmelo.petronio@uniroma1.it>, Luca Pandolfi <luca.pandolfi@uniroma3.it>

Egregio dott. Marra
La prego di concordare la data con il dott. Luca Bondioli, che legge p.c., responsabile del laboratorio. Lo contatti per email all'indirizzo luca.bondioli@beniculturali.it poiché fino al 9 maggio non sarà in servizio

dott. Francesco RUBAT BOREL, PhD
direttore del Museo Nazionale Preistorico Etnografico "Luigi Pigorini"
piazza Guglielmo Marconi, 14
I-00144 Roma, Italia
tel. +39 06.54.95.22.35 (centralino +39 06.54.95.21)
E-mail francesco.rubatborel@beniculturali.it
www.pigorini.beniculturali.it
fax +39 06.54.95.23.10

Polo Museale del Lazio
piazza San Marco, 49
I-00186, Roma, Italia
tel. +39 06.69.99.42.51 - 06.69.99.43.42
E-mail pm-laz@beniculturali.it
PEC mbac-pm-laz@mailcert.beniculturali.it
www.polomusealelazio.beniculturali.it

Da: fabrizio marra [fabrizio.marra@ingv.it]
Inviato: mercoledì 27 aprile 2016 11.26
A: RUBAT BOREL FRANCESCO; GABRIELLI EDITH
Cc: leonardosalari@virgilio.it salari; Carmelo Petronio; Luca Pandolfi
Oggetto: rinnovo richiesta autorizzazione

Egr. Dottor Rubat Borel,
facendo seguito alla e-mail del 2 marzo, in cui ho inviato copia della richiesta di autorizzazione di poter prendere visione dei resti fossili provenienti dall'Area di Saccopastore (Roma) conservati presso il museo Pigorini, e alla successiva del 18 aprile scorso, alle quali non abbiamo ricevuto risposta, le chiedo gentilmente di voler indicare una possibile data per effettuare l'analisi del materiale, per la quale l'INGV si affida al Professor Carmelo Petronio e ai dottori Leonardo Salari e Luca Pandolfi, che leggono in copia.
La ringrazio per la cortese collaborazione e le porgo cordiali saluti,

Fabrizio Marra

INGV
terremoti
vulcani
ambiente
ISTITUTO NAZIONALE
DI GEOFISICA E VULCANOLOGIA

Rome, November 21, 2016

to: Fabrizio Marra

Primo Ricercatore
Istituto Nazionale di Geofisica e Vulcanologia
Via di Vigna Murata 605, 00143, Roma
tel. 06 51860420
fax 06 51860507
fabrizio.marra@ingv.it

Dear Dr. Marra

you have my permission to use and modify if necessary the Digital Elevation

Map (DEM) for the region of Rome (WA 6570) for your research.

Dr. Fabio Florindo

Director, Environment Department
Istituto Nazionale di Geofisica e Vulcanologia
Rome - Italy
fabio.florindo@ingv.it

Sezione RM1
Sismologia e Tettonofisica

Via di Vigna Murata, 605

00143 ROMA | Italia
Tel.: +39 06518601
Fax: +39 0651860507
aoo.roma1@pec.ingv.it
www.roma1.ingv.it

Patrizia Gioia, Director
Museo di Casal de' Pazzi
via Ciciliano, s.n.c. incrocio via Egidio Galbani, Roma, Italy

Rome, 11-24, 2016

I have authorized the survey and the reproduction under the specific Creative Commons Attribution License (CCAL), CC BY 4.0. in PLos One of the photography taken by Fabrizio Marra at Museo Casal de' Pazzi.

Yours Sincerely

Patrizia Gioia

Giuseppe Gisotti, Director
Società Italiana di Geologia Ambientale (SIGEA)
Casella Postale 2449 U. P. ROMA 158
(via Marsala 39 – 00185 Roma)
Tel 06.5943344
E-mail: info@sigeaweb.it; Web: www.sigeaweb.it

Rome, 11-20, 2016

SIGEA authorizes the reproduction under the specific Creative Commons Attribution License (CCAL), CC BY 4.0. in Plos One of the photograph from the book "I geositi del territorio di Roma Capitale" showing an outcrop in Via dei Prati Fiscali, Rome.

Yours Sincerely,

Giuseppe Gisotti
