# Supplementary figures and images for: The Aggradational Successions of the Aniene River Valley in Rome: Age Constraints to Early Neanderthal Presence in Europe
Source: PLoS One. 2017 Jan 26;12(1):e0170434. doi: 10.1371/journal.pone.0170434 (PMC5268786; doi:10.1371/journal.pone.0170434)

**Supplementary Online Material - Casal de' Pazzi stratigraphic logs**

S-347


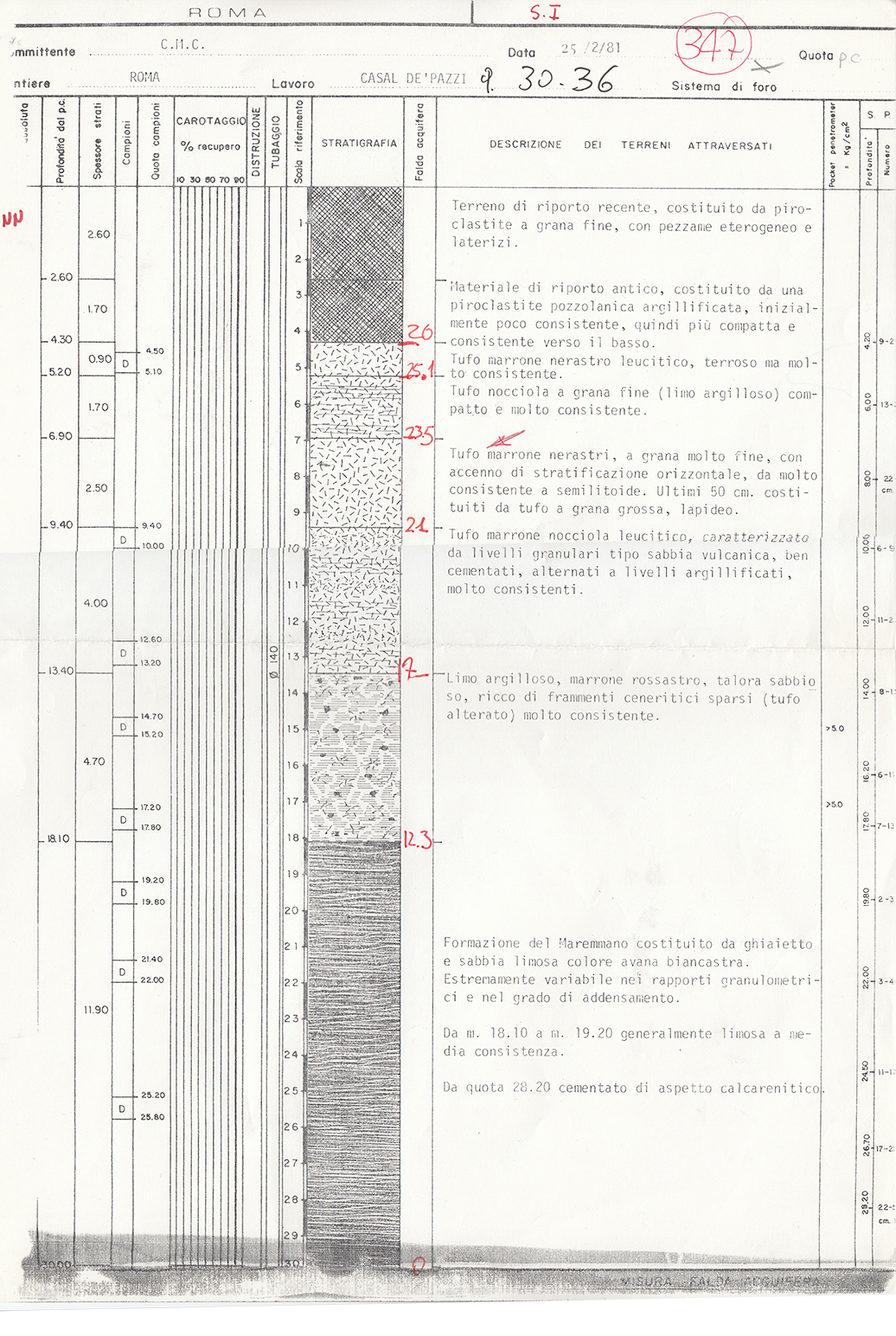


S-497


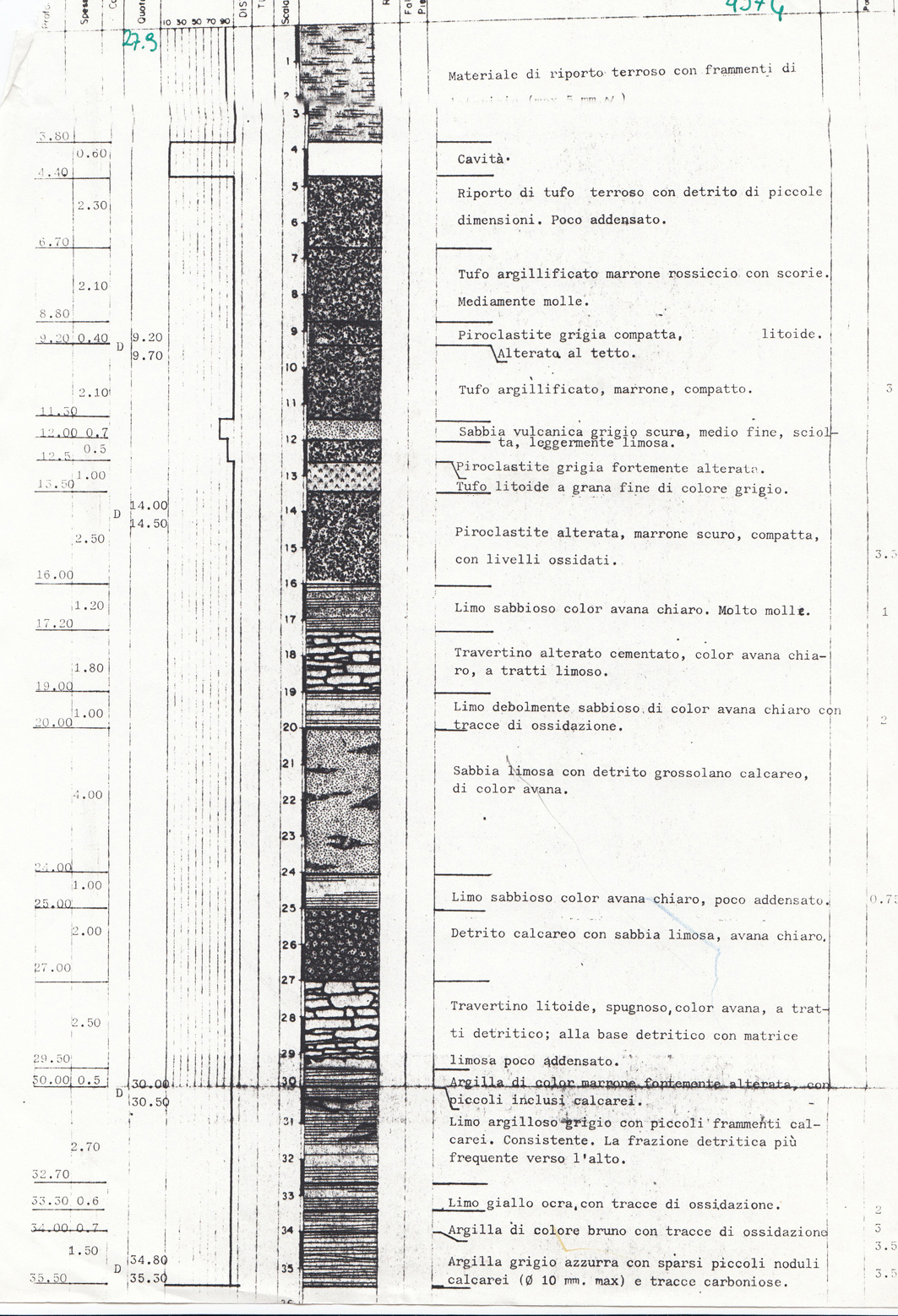

Supplement: S2 File — (DOC) [file pone.0170434.s002.doc]
